# Bridging nutrition and neurology: malnutrition's role in perioperative neurocognitive disorders

**DOI:** 10.3389/fnut.2025.1601021

**Published:** 2025-08-13

**Authors:** Li Luo, Qihai Gong, Miao He, Yuhang Zhu, Wanqiu Yu, Taowu Gong, Pengcheng Zhao, Zhaoqiong Zhu

**Affiliations:** ^1^Anesthesia Department, Affiliated Hospital of Zunyi Medical University, Zunyi, Guizhou, China; ^2^Anesthesia Department, Suining Central Hospital, Suining, Sichuan, China; ^3^School of Pharmacy, Key Laboratory of Basic Pharmacology of Ministry of Education and Joint International Research Laboratory of Ethnomedicine of Ministry of Education, Zunyi Medical University, Zunyi, Guizhou, China; ^4^Anesthesia Department, Affiliated Hospital of Chengdu University, Chengdu, Sichuan, China; ^5^Anesthesia Department, The First People's Hospital of Zunyi, Zunyi, Guizhou, China; ^6^Early Clinical Research Ward, Affiliated Hospital of Zunyi Medical University, Zunyi, Guizhou, China

**Keywords:** perioperative neurocognitive disorders (PND), malnutrition, cognitive dysfunction, nutritional intervention, gut-brain axis

## Abstract

This literature review examines the relationship between malnutrition and perioperative neurocognitive disorders (PND), which encompass cognitive impairments occurring throughout the perioperative period, including pre-existing cognitive impairments, postoperative delirium, delayed neurocognitive recovery, and postoperative cognitive dysfunction. Malnutrition is associated with an increased incidence of PND, affecting patient recovery and quality of life. Studies suggest that preoperative malnutrition may heighten the risk of PND, and that preoperative nutritional diagnosis and perioperative nutritional interventions could reduce the occurrence of PND. The review discusses the definition, diagnosis, and indicators of malnutrition, as well as the mechanisms by which malnutrition leads to PND, including direct pathways such as psychological factors, abnormal neurotransmitter synthesis, and changes in brain structure and function, and indirect pathways like impaired immune function, neuroinflammation, mitochondrial dysfunction, intestinal barrier damage, disruption of the gut-brain axis, lymphatic system dysfunction, and endocrine disruption. Finally, this paper summarizes the existing nutritional intervention strategies for improving PND, explores the research directions of malnutrition and PND, and emphasizes that future research needs to clarify the role of nutritional intervention in specific populations and conduct in-depth studies on the molecular mechanisms of nutritional intervention and PND prevention.

## 1 Introduction

**Figure 1:**
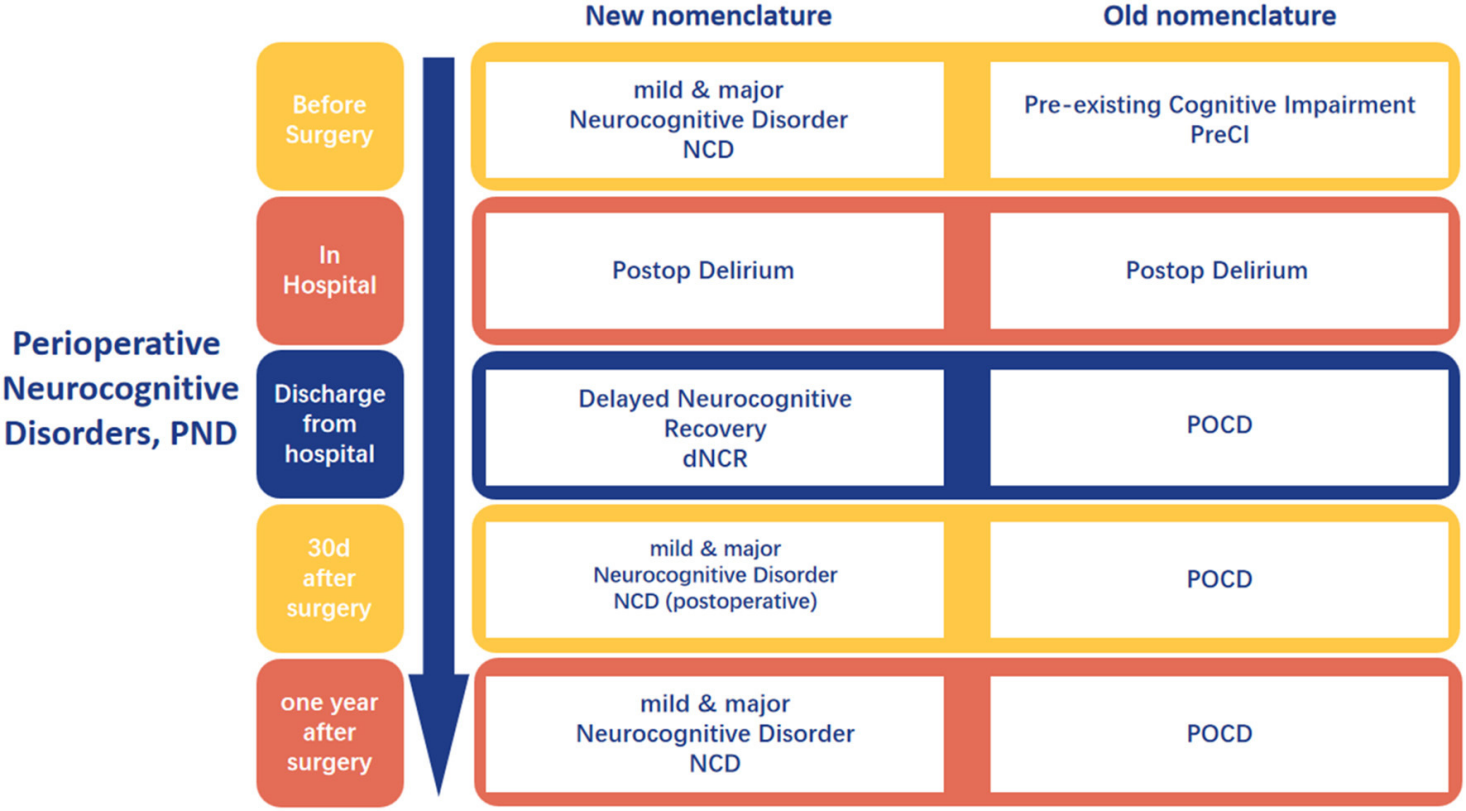
The nomenclature about the perioperative neurocognitive disorder. Created with FigDraw.com.

Perioperative neurocognitive disorders (PND) encompasses cognitive impairment or decline that occurs throughout the perioperative period, manifesting as deficits in orientation, attention, memory, and reaction time (1). This includes pre-existing cognitive impairments diagnosed preoperatively, delirium occurring during the postoperative hospital stay postoperative delirium (POD), delayed neurocognitive recovery diagnosed within 30 days postoperatively delayed neurocognitive recovery (dNCR), and persistent or diagnosed cognitive decline within 12 months postoperatively, classified as severe or mild neurocognitive dysfunction postoperative cognitive dysfunction (POCD) (Figure 1) (2).

PND is a widely recognized public health issue that may affect millions of patients annually (1). It is associated with increased postoperative mortality, increased complications, prolonged hospital stays, and higher medical costs (3). Furthermore, long-term follow-up studies suggest that patients with PND are at an increased risk of dementia, reduced quality of life, and diminished work capacity (4).

Studies indicate that various perioperative risk factors may be associated with the development of PND, including advanced age, surgical site, type of surgery, duration of surgery, intraoperative blood loss, hypotension and higher ASA physical status classes (5); preoperative comorbidities such as hypertension, diabetes, obesity, sleep disorders, presence of depressive, anxiety states, and dementia (6); history of delirium, alcohol abuse, and substance use, postoperative sleep disturbances, inadequate pain management, postoperative infections, repeated use of anesthetics (7).

In recent years, the nutritional status has gained increasing research attention, with up to one-third of patients presenting with malnutrition or being at risk of malnutrition upon admission (8). Causes include disease-related anorexia, medication-related side effects, fasting orders related to diagnostic procedures, diseases that impair normal gastrointestinal function, suboptimal overall nutritional management in hospitalized patients, and metabolic consumption caused by diseases (9, 10). These conditions can lead to decreased appetite or impaired nutrient absorption.

Recent research on the relationship between malnutrition and PND has highlighted the significant association between the two (11). Numerous studies have demonstrated that preoperative malnutrition may increase the risk of PND, and that preoperative nutritional diagnosis and perioperative nutritional interventions may be beneficial in reducing the incidence of PND (12). Early identification and intervention of malnutrition are expected to effectively reduce the incidence of PND, improve the quality of patient recovery, and enhance postoperative quality of life (13). This review of the literature on malnutrition leading to perioperative neurocognitive disorders aims to provide a multifaceted perspective for the mechanistic study of PND.

To conduct this narrative review, we searched the following databases: PubMed, Web of Science, and Scopus. The search was performed using the keywords (“Malnutrition”[Mesh] OR “Nutrition Disorders”[Mesh] OR “Nutritional Status”[Mesh] OR “Nutrition Assessment”[Mesh] OR “Nutritional Support”[Mesh] OR “Nutrition Therapy”[Mesh] OR “Nutritional Deficiency”[Mesh] OR “Protein-Energy Malnutrition”[Mesh] OR “Undernutrition” OR “Nutrition Risk” OR “Nutritional Risk” OR “Nutritional Screening” OR “Malnourished” OR “Nutritional Inadequacy” OR “Nutritional Depletion”) AND (“Perioperative Period”[Mesh] OR “Perioperative Care”[Mesh] OR “Surgical Procedures, Operative”[Mesh] OR “Anesthesia”[Mesh] OR “Anesthesia Recovery Period”[Mesh] OR “Postoperative Period”[Mesh] OR “Preoperative Period”[Mesh] OR “Intraoperative Period”[Mesh] OR “Surgery” OR “Surgical” OR “Operative” OR “Anesthesia” OR “Anesthetic” OR “Preoperative” OR “Intraoperative” OR “Postoperative” OR “Post-surgery” OR “Post-surgical” OR “Perioperative”) AND (“Cognition Disorders”[Mesh] OR “Cognitive Dysfunction”[Mesh] OR “Postoperative Cognitive Complications”[Mesh] OR “Delirium”[Mesh] OR “Confusion”[Mesh] OR “Neurocognitive Disorders”[Mesh] OR “Postoperative Cognitive Dysfunction” OR “POCD” OR “Postoperative Cognitive Decline” OR “Postoperative Delirium” OR “Cognitive Impairment” OR “Cognitive Decline” OR “Neurocognitive Decline” OR “Cognitive Deficit” OR “Mental Status Change”)and was limited to articles published in English between January 2010 and January 2025. Foundational studies published prior to 2010 were included where critical to contextualize key concepts. We included original research articles, reviews, and case studies that focused on “perioperative cognitive dysfunction caused by malnutrition” and excluded articles that were not relevant to the main topic, duplicates, and those with insufficient data. The articles were screened based on their titles and abstracts, and full texts were retrieved for those that met the inclusion criteria. The key findings and conclusions were extracted and synthesized into major themes for discussion in this review.

## 2 Malnutrition definition, diagnosis, and indicators

**Figure 2:**
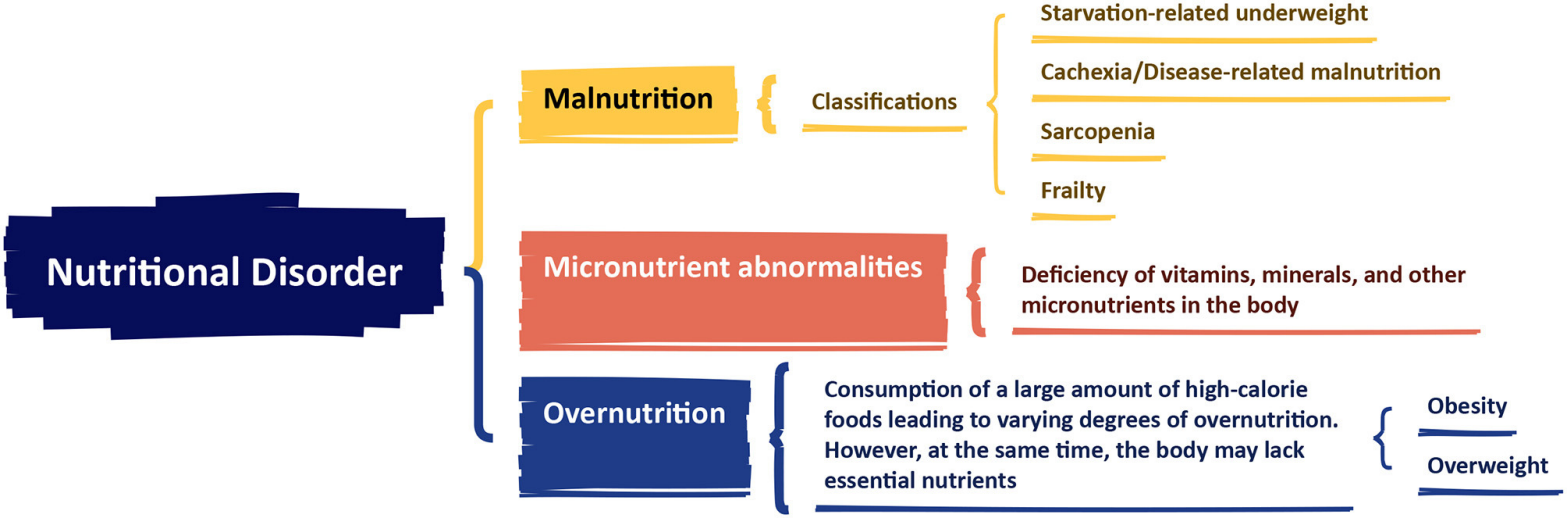
The European Society for Clinical Nutrition and Metabolism (ESPEN) consensus statement on malnutrition. Created with FigDraw.com.

In 2015, the European Society for Clinical Nutrition and Metabolism (ESPEN) released a consensus statement on malnutrition, defining it as: “Malnutrition is a condition resulting from a lack of food intake or absorption, leading to changes in body composition that affect body and mental function and impact clinical outcomes of disease” (14). The statement introduced the concept of nutrition disorder and its diagnostic framework, categorizing nutrition disorders into three types: Malnutrition, Micronutrients abnormalities, and Overnutrition. Malnutrition was further divided into four subtypes: Starvation-related underweight, Cachexia/Disease-related malnutrition, Sarcopenia, and Frailty (Figure 2). In 2018, the Global Leadership Initiative on Malnutrition (GLIM), a group of global nutrition leaders, launched the latest criteria for malnutrition assessment, simply referred to as the GLIM criteria (15). The GLIM criteria clearly distinguish between two steps: “nutrition screening” and “diagnostic assessment” (16). The first step is nutrition screening, which emphasizes the use of clinically validated nutrition screening tools for patient assessment. The criteria list the Nutritional Risk Screening (NRS 2002) (17), Malnutrition Universal Screening Tool (MUST) (18), and Mini Nutritional Assessment-Short Form (MNA-SF) (19) as the primary screening tools. The second step, following a positive screen, is to assess and grade the severity of malnutrition. The malnutrition assessment criteria include five items: involuntary weight loss, low BMI, and reduced muscle mass, which are phenotypic criteria; and reduced food intake or absorption, and disease burden/inflammation, which are etiologic criteria (15). To make a diagnosis of malnutrition, at least one phenotypic diagnostic criterion and one etiologic diagnostic criterion must be met. Based on the degree of weight loss, low BMI, and muscle wasting, malnutrition is classified as moderate malnutrition (Phase 1) and severe malnutrition (Phase 2) (Table 1).

**Table 1:** The criteria for malnutrition assessment from Global Leadership Initiative on Malnutrition (GLIM).

**Malnutrtion**				
Standard	Phenotypic criteria	①Involuntary weight loss	②low BMI	③Decreased muscle mass
		Meets at least one criterion		
	Etiologic criteria	④Reduced food intake or absorption	⑤Disease burden/inflammation	
		Meets at least one criterion		
Stages	Stage 1: Moderate malnutrition	①Weight loss: Lose 5%−10% within 6 months, or 10%−20% after 6 months	②Low BMI: <20 kg/m^2^ under 70 years of age, or <22 kg/m^2^ over 70 years of age	③Decreased muscle mass: Mild to moderate reduction
		Meets at least one criterion		
	Stage 2: Severe malnutrition	①Weight loss: Loss >10% within 6 months, or >20% after 6 months	②Low BMI: <18.5 kg/m^2^ under 70 years of age, or <20 kg/m^2^ over 70 years of age	③Decreased muscle mass: Reduction in severity
		Meets at least one criterion		

Currently, commonly used nutritional indicators in clinical and research settings include Albumin (ALB), Prognostic Nutritional Index (PNI), and Geriatric Nutritional Risk Index (GNRI).

Plasma ALB is influenced by various factors (such as infection, blood loss, severe hepatic or renal dysfunction, etc.), and its ability to assess nutritional risk is controversial (20). Nevertheless, plasma ALB at admission or before surgery is still used as an indicator to assess patient nutritional risk in clinical practice. PNI is a comprehensive indicator for assessing the nutritional status of surgical patients, predicting surgical risks, and making prognostic judgments. It reflects the immune-nutritional condition by calculating the function of peripheral blood lymphocyte counts and serum albumin (21). In a study related to spinal deformity correction surgery, it was found (22). The significant risk factors for POD were age (OR 1.11, 95%CI 1.03–1.19) and PNI (OR 0.87, 95%CI 0.79–0.96). The risk factors for POD after spinal deformity correction are PNI <49.7 and age > 68.5 years old. GNRI is a simple and objective indicator for assessing the nutritional status of elderly patients and can be used to predict the risk of nutrition-related complications and death (23). The calculation of GNRI involves weight, height, and serum albumin levels, with the value range reflecting the patient's nutritional status, such as 97.5–100 indicating mild malnutrition, 83.5–97.5 indicating moderate malnutrition, and <83.5 indicating severe malnutrition (24).

## 3 Malnutrition leading to the occurrence of PND

### 3.1 The influence of different types of malnutrition on PND

#### 3.1.1 Energy-related malnutrition (ERM) and PND

A pathological condition specifically caused by prolonged energy-protein intake deficiency, characterized by energy imbalance (chronic intake below basal metabolic requirements) and protein deficiency (<0.8 g/kg/day). ERM is commonly seen in cases of insufficient intake, absorption disorders, or a hypermetabolic state of the body caused by disease-related reasons. ERM increases PND risk through three mechanisms: ① Inflammatory cascade: Hypoproteinemia triggers IL-6 and TNF-α release, disrupting the blood-brain barrier and directly damaging neurons (25); ② Neurotransmitter imbalance: Protein deficiency leads to tryptophan metabolism disorder and accumulation of neurotoxic quinolinic acid (26); ③ Cerebral energy crisis: Insufficient glucose supply forces neurons to rely on ketones, exacerbating oxidative damage through mitochondrial dysfunction (27). Relevant studies demonstrate: 79.3% of elderly hip fracture patients exhibit ERM, with a 2.3-fold increased risk of postoperative delirium (OR = 3.4, 95%CI 1.9–6.1) (28); Cardiac surgery patients with GNRI <92 show a 3.5-fold increased PND incidence (23); Intervention studies confirm that 7-day preoperative high-protein supplementation (≥1.5 g/kg/day) combined with vitamin D (2,000 IU/day) reduces PND risk by 48% (29). In conclusion, ERM represents a modifiable key risk factor for PND, with early nutritional screening and targeted protein supplementation potentially improving neurocognitive outcomes.

#### 3.1.2 Sarcopenia and PND

It is manifested as muscle strength reduction and muscle mass decline, and may be accompanied by a decline in physical function. Increasing the intake of protein and vitamins can prevent and alleviate sarcopenia. Relevant studies have shown (30) that the prevalence of sarcopenia ranges from 18% in diabetic patients to 66% in patients with inoperable esophageal cancer. Sarcopenia is associated with a high risk of various adverse health outcomes, including poor overall survival and disease-free progression survival rates, postoperative complications, prolonged hospital stays for patients with different medical conditions, as well as falls and fractures, metabolic disorders, cognitive impairment, and mortality in the general population. In a prospective cohort study on the impact of sarcopenia on cognitive function in 2015 (31), a total of 5,715 participants over 60 years old (43.8% were female; average age 67.3 ± 6.0 years) participated in a cross-sectional association study, and 2,982 elderly people were followed up in 2018. Logistic regression models showed that compared with the non-sarcopenia group, the OR value of the possible sarcopenia group was 1.43 (95% CI: 1.06–1.91, *P* = 0.017), and the OR value of the sarcopenia group was 1.72 (95% CI: 1.04–2.85, *P* = 0.035). Sarcopenia is associated with more severe cognitive impairment, providing new evidence for a strong association.

#### 3.1.3 Frailty and PND

Frailty is a non-specific state characterized by a decline in the physiological reserves of the nervous, muscular, metabolic and immune systems, increased vulnerability of the body, and reduced stress resistance. Some people, although they do not have specific diseases, are prone to fatigue, weakness and weight loss. The imbalance between energy supply and demand related to nutrition and the disorder of the internal environment may be one of the reasons. In existing studies, there are many reports on the association between frailty and PND. Cheng et al. (32) found in a recent cohort study of 2,080 elderly people that the incidence of delirium was higher in the frailty group (29.2% vs. 16.4%, *P* < 0.05). After adjusting for related variable factors, multivariate logistic regression showed that the risk of delirium in frailty patients was significantly increased (adjusted OR: 1.61, 95% CI: 1.23–2.10, *P* < 0.001, E value: 1.85). In addition, in a prospective observational study in Taiwan (33), frailty was found to be an independent risk factor for POD in elderly cancer patients after elective abdominal surgery. In another meta-analysis of cohort studies (34), a total of 15 cohort studies were included, involving 3,250 adult patients who underwent surgery. The preoperative prevalence of frailty was 27.1% (880/3,250). The pooled results showed that frailty was significantly associated with a higher risk of POD (adjusted OR: 3.23, 95% CI: 2.56–4.07, *P* < 0.001).

#### 3.1.4 Micronutrients abnormalities and PND

Malnutrition caused by a deficiency of micronutrients such as vitamins and minerals. In a systematic review on micronutrient deficiency and POD (35), it was found that micronutrient deficiency (i.e., cobalamin, thiamine, and vitamin D) was associated with an increased incidence of delirium, with a higher prevalence among hospitalized patients. In a prospective cohort study on vitamin D and the occurrence of delirium after coronary artery bypass grafting (36), multivariate logistic regression analysis indicated that severe vitamin D deficiency at admission was associated with the occurrence of delirium (OR: 3.18; 95% CI: 1.29–7.78; *P* = 0.01). A 2022 meta-analysis on preoperative vitamin deficiency and POD and cognitive dysfunction (37) also reached the same conclusion, that preoperative vitamin D deficiency was associated with postoperative cognitive impairment. The results of a systematic review showed (38) that low levels of B vitamins (folic acid and vitamin B12), vitamin D, vitamin A, vitamin E, omega-3 fatty acids, and albumin, as well as high levels of homocysteine in the blood, were significantly associated with an increased risk of mild cognitive impairment in the elderly.

Beyond classical micronutrients, dietary and non-dietary antioxidants play pivotal roles in mitigating oxidative stress-induced neuronal damage, a key pathway in PND pathogenesis. Evidence suggests that nutritional antioxidants (e.g., vitamin C, selenium) enhance endogenous defense systems by scavenging reactive oxygen species (ROS) and supporting glutathione peroxidase activity (39, 40). Phenolic compounds (e.g., resveratrol, curcumin) exert neuroprotection via anti-inflammatory signaling (e.g., NF-κB suppression) and mitochondrial biogenesis regulation (e.g., PGC-1α activation) (41, 42). Clinical studies report that perioperative supplementation with these antioxidants correlates with reduced PND incidence, likely through preserving blood-brain barrier integrity and synaptic function (43).

#### 3.1.5 Overnutrition and PND

Obesity represents a distinct form of malnutrition characterized by chronic oxidative stress and micronutrient deficiencies, even amidst caloric excess. Although obese people consume excessive calories, they often have insufficient intake or absorption of nutrients such as vitamins (like B vitamins, D, and E) and minerals (such as iron, zinc, and magnesium), presenting a state of “energy surplus but nutritional imbalance”. At the same time, a preference for high-sugar, high-fat and high-oil foods leads to excessive calorie intake, while lacking key nutrients such as dietary fiber and high-quality protein, which affects metabolism. In addition, obese individuals are prone to insulin resistance, abnormal leptin secretion, and other conditions, which lead to fat accumulation and nutrient utilization disorders, creating a vicious cycle. High intake of processed foods rich in saturated fats (e.g., palmitic acid) and refined sugars—hallmarks of the Western Diet—induces mitochondrial dysfunction and ROS overproduction in the brain (44). This persistent oxidative state exacerbates neuroinflammation and blood-brain barrier disruption, significantly elevating POD risk beyond isolated micronutrient deficiencies (45). Clinical evidence confirms this linkage: Feinkohl et al. (46) reported a 1.85-fold higher POD risk (95% CI: 1.26–2.70) in obese patients with metabolic syndrome, where Western Diet patterns drive systemic oxidation. In overweight hip fracture patients, malnutrition (often coexisting with obesogenic diets) increased POD incidence (OR = 3.64) (47). Crucially, the Western Diet's impact on POD is amplified by: ① Deficiencies in antioxidant cofactors (zinc, selenium) required for ROS-detoxifying enzymes (e.g., SOD). ② Low intake of phenolic compounds (e.g., flavonoids in berries), which synergistically regulate Nrf2/NF-κB pathways (48). This is consistent with our emphasis in the previous section on dietary antioxidants as a response to PND.

### 3.2 The influence of malnutrition on PND in different surgical types

Although the degree of trauma and stress response varies among different types of surgery, the correlation between malnutrition and the occurrence of PND after surgery appears to be well-established. A study from West China Hospital of Sichuan University assessed preoperative nutritional status using GNRI and MNA-SF and followed up with patients for the occurrence of POD and length of hospital stay (LOS), finding that preoperative malnutrition is significantly associated with POD, and that low/high nutritional risk of preoperative GNRI and malnutrition of MNA-SF are independent predictive factors for prolonged LOS (49). Wang et al. (50) found in a retrospective study that among 1,440 patients who underwent hip fracture surgery, the incidence of POD was 19.1%, and the risk of POD in patients with hypoalbuminemia increased by 2.99 times, with a dose-response relationship between low albumin levels and POD risk. Hung et al.'s (51) meta-analysis found a negative correlation between PNI and POD, with the risk of POD nearly doubling. Chen et al. (23) explored the predictive value of GNRI for POD in elderly cardiac surgery patients and found that malnourished patients (GNRI ≤ 98) had a significantly higher risk of POD compared to non-malnourished patients (GNRI > 98), with a negative correlation between preoperative GNRI and POD. Another study found that a lower GNRI increases the risk of delirium and dementia and is a risk factor for death within 1 year after hip surgery (24). In Mazzola's study, elderly patients who underwent hip fracture surgery and were malnourished or severely malnourished were more likely to experience POD (52). A prospective observational study found that 44.5% of patients experienced POD, with advanced age, hypoalbuminemia, malnutrition, and uncontrolled diabetes being strong predictive factors for POD in elderly hip fracture patients (53). A prospective cohort study using the National Hip Fracture Database (NHFD) also found that preoperative cognitive deficits and malnutrition, among other factors, were associated with POD (54).

## 4 Mechanisms of malnutrition leading to PND

### 4.1 Direct pathways

#### 4.1.1 Psychological factors and preoperative cognitive function

Patients with malnutrition often have poor physical conditions, which can lead to adverse emotions such as anxiety and depression, affecting their sleep quality and psychological state, thereby increasing their susceptibility to PND. A survey study based on the NHANES database analyzed the association between the GNRI and the prevalence and scores of depression, indicating that the GNRI in the depressed group was significantly lower than in the non-depressed group (55). Multivariate logistic regression showed that GNRI is an important predictor of depression. Depression and anxiety are correlated with cognitive decline that depression is a major risk factor for dementia and mild cognitive impairment (56). If patients already have preoperative cognitive decline, their brain function may be further challenged after surgery and anesthesia, potentially exacerbating cognitive dysfunction and leading to POD.

The pathophysiological links between malnutrition and mood disorders involve specificmicronutrient deficiencies. Notably, deficiencies in B vitamins (folate/folic acid, B6, B12) impair one-carbon metabolism, elevating homocysteine while reducing S-adenosyl methionine (SAM) (57). As SAM is the methyl donor for synthesizing serotonin, dopamine, and norepinephrine (58), its depletion in malnourished patients directly disrupts neurotransmitter homeostasis—a mechanism corroborated by lower SAM levels in depressed individuals (59). Concurrently, excess homocysteine promotes neurotoxicity via overactivation of NMDA receptors (60), further exacerbating depressive pathophysiology.

Simultaneously, broader nutritional factors modulate anxiety pathways. Key nutrients (B vitamins, vitamin C, magnesium, zinc) regulate stress responses through neurotransmitter synthesis/metabolism and neuronal membrane stability (61). Chronic stress impairs these processes, creating a vicious cycle that heightens anxiety risk. Furthermore, these nutrients facilitate conversion of α-linolenic acid (ALA) to neuroprotective n-3 fatty acids (62), which are independently associated with reduced anxiety. Collectively, these mechanisms elucidate how malnutrition propagates emotional dysregulation, ultimately predisposing to PND.

#### 4.1.2 Abnormal synthesis of neurotransmitters

Neurotransmitters, as key messengers in the transmission of nervous system information, rely on various nutrients for their synthesis. Tryptophan and tyrosine, as precursors of serotonin and dopamine, respectively, are insufficiently ingested during malnutrition, particularly under conditions of prolonged hunger or energy deficit. Crucially, in such states, the body may prioritize these amino acids for energy production or gluconeogenesis over neurotransmitter synthesis, further limiting their availability for neural signaling. This leads to a significant decrease in the activity of enzymes involved in the synthesis of these neurotransmitters, resulting in the obstruction of neurotransmitter synthesis, an imbalance in neurotransmitter levels, and subsequently affects the brain's emotional regulation and cognitive functions, increasing the risk of POD (63). Importantly, this neurotransmitter imbalance, especially involving serotonin and dopamine which are key regulators of appetite, mood, and motivation, can disrupt hypothalamic feeding centers and reward pathways. This disruption may manifest as altered appetite perception, reduced motivation to seek food, or changes in metabolic set points, thereby exacerbating the initial malnutrition and perpetuating a detrimental cycle of nutritional deficiency and neurological dysfunction. Neurotrophic B vitamins also play a crucial role in neurotransmitter metabolism and the maintenance of a healthy nervous system, particularly vitamins B1, B6, and B12. Studies have shown that vitamin B12 can assist in the synthesis of methylmalonyl-CoA, and a deficiency in this coenzyme can lead to impaired myelin formation, thereby disrupting the conduction of nerve impulses (64).

#### 4.1.3 Changes in brain structure and function

Long-term malnutrition can lead to significant changes in brain structure and function. Neuroimaging examinations suggest that in children with malnutrition, MRI results mainly show brain atrophy, which tends to be reversible after appropriate nutritional intervention (65). Elderly patients with malnutrition have a higher incidence of white matter hyperintensities compared to those in good nutritional status, and the study also indicates that low levels of vitamins B1 and B12 increase the risk of white matter hyperintensities (66). Another study shows that vitamin D deficiency in the elderly is associated with increased white matter hyperintensities in periventricular, cortical, and juxtacortical regions, as well as atrophy of the hippocampus, anterior cingulate cortex, and left calcarine sulcus gray matter (67). Animal studies suggest that in mice deficient in vitamin A, there are changes in the proteome of the cerebral cortex and hippocampus (68). These structural and functional abnormalities affect the brain's information transmission and integration, greatly increasing the risk of postoperative cognitive dysfunction and delirium symptoms in patients.

### 4.2 Indirect pathways

#### 4.2.1 Impaired immune function and neuroinflammation

Malnutrition, encompassing both undernutrition (insufficient intake) and overnutrition (excess intake leading to overweight/obesity), can lead to significant immune dysfunction, albeit through distinct mechanisms. In the context of undernutrition, there is a comprehensive impairment of immune function, characterized by significant reductions in the function of immune cells such as macrophages and T-cells, and an imbalance in their proportions (69). Perioperative patients with undernutrition have a weakened ability to resist external pathogens, resulting in a significantly increased incidence of infections (70). Conversely, in individuals with overnutrition, such as those who are overweight or obese, and particularly those with associated comorbidities like metabolic syndrome, diabetes, and hypertension, a state of chronic low-grade inflammation (metaflammation) is prevalent even in the absence of overt infection. This state is characterized by persistently elevated levels of pro-inflammatory cytokines, including interleukin-1β (IL-1β), interleukin-6 (IL-6), and tumor necrosis factor-α (TNF-α).

Regardless of the underlying cause (infection in undernutrition or chronic metaflammation in overnutrition), elevated levels of circulating inflammatory factors pose a risk to the central nervous system. These cytokines can partially enter the brain through the weak areas of the blood-brain barrier, and another part achieves transmembrane transport by activating transport proteins on endothelial cells (71, 72). Noah et al. (73), in a meta-analysis, explored the association between preoperative blood levels of inflammatory mediators and POD by assessing these levels before surgery. The results indicated that participants who developed POD had significantly higher preoperative IL-6 levels compared to those who did not, suggesting that a pre-existing inflammatory state (whether from infection, chronic disease, or nutritional imbalance) may increase the risk of POD.

After infiltrating the brain, inflammatory factors rapidly activate microglia, the innate immune cells of the central nervous system, transforming them from a resting state to an activated state with phagocytic and antigen-presenting functions, releasing more inflammatory mediators and neurotoxic substances, such as reactive oxygen species (ROS) and nitric oxide (NO) (74). The activation of microglia is often considered a key contributor to POCD, as it involves the interaction between neuroinflammation and neurofunctional abnormalities, neuronal apoptosis, and synaptic damage, leading to neurologic dysfunction (75, 76). In addition to the excessive production of inflammatory cytokines and reactive oxygen species, other perioperative injury factors, such as brain iron metabolism imbalance and downregulation of type 2 cannabinoid receptors, can also induce microglial activation (77, 78). Activated microglia form a positive feedback loop with injury factors, exacerbating the damage process and ultimately leading to postoperative neurological sequelae.

#### 4.2.2 Mitochondrial dysfunction

The brain is one of the most metabolically active organs in the human body, primarily dependent on the oxidation of glucose and fatty acids for energy supply. Mitochondria, as cellular organelles of energy metabolism, are important sites for the complete oxidation of glucose or its transformation into substances like fatty acids. In states of malnutrition, deficiencies in vitamins or trace elements affect the activity of key cofactors like Mg^2+^ in glycolytic enzymes such as hexokinase and pyruvate kinase, leading to reduced efficiency in glucose uptake and utilization (79). In addition to energy supply, mitochondria play a significant role in the generation of reactive oxygen species, cell death, and Ca^2+^ buffering. Under normal physiological conditions, mitochondria produce a small amount of reactive oxygen to participate in intracellular signal transduction, while mitochondrial dysfunction can lead to the production of large amounts of reactive oxygen, triggering oxidative stress damage and contributing to neurological diseases (80). Mitochondria are also involved in various forms of cell death, including apoptosis, pyroptosis, ferroptosis, and cuproptosis (81, 82). Furthermore, mitochondria play a crucial role in maintaining intracellular calcium ion homeostasis, which is particularly important for excitable cells like neurons (83). A substantial body of evidence suggests that mitochondrial dysfunction can lead to hippocampal neuron damage and is a key factor in triggering cognitive impairment in PND (84, 85). Improving mitochondrial dysfunction in elderly mice with abdominal exploration can inhibit oxidative stress, insufficient energy supply, and mitochondrial ultrastructural abnormalities (86). In PND mice, glucose metabolism disruption mediated by glucose transporter 1 and reduced ATP production due to mitochondrial dysfunction have been observed (87). Isoflurane anesthesia in mice leads to cognitive impairment, opening of the mitochondrial permeability transition pore in hippocampal tissue, and a decrease in ATP levels and mitochondrial membrane potential. In the isoflurane/surgery-induced cognitive impairment model, a decrease in basal oxygen consumption, ATP levels, and maximum mitochondrial respiratory capacity in the hippocampus of mice has been noted (88). Isoflurane can cause energy supply deficiency in the brain, leading to a reduction in postsynaptic density protein 95 in hippocampal tissue, inhibition of the brain-derived neurotrophic factor/tyrosine kinase receptor B pathway, synaptic dysfunction, and ultimately learning and memory impairments (89). It is evident that mitochondrial energy metabolism disorders leading to insufficient brain energy supply are one of the mechanisms promoting the occurrence of PND.

#### 4.2.3 Damage to the intestinal barrier and disruption of the gut-brain axis

In recent years, the gut microbiome-gut-brain axis has become an important field in biological research. The gut, as the largest immune and nutrient-absorbing organ in the human body, sees its mucosal barrier integrity compromised and intestinal permeability increased under conditions of malnutrition, allowing the translocation of bacterial endotoxins and pathogens into the bloodstream (90). At the same time, malnutrition causes an imbalance in the gut microbiota, with a reduction in beneficial bacteria (lactobacilli, bifidobacteria) increasing the risk of postoperative cognitive impairment, and surgical anesthesia exacerbates the ecological imbalance of the gut microbiota, shifting it toward a more toxic phenotype (91). Other perioperative factors also affect the gut microbiota, including antibiotics, opioids, or acidifying drugs (92).

A critical aspect of gut-brain communication involves serotonin (5-hydroxytryptamine, 5-HT). Notably, approximately 90% of the body's serotonin is produced in the gut, primarily by enterochromaffin (EC) cells lining the intestinal epithelium (93). This peripheral serotonin plays a vital role not only in regulating gut motility and secretion (94), but also, crucially, in modulating mood, cognition, and stress responses via the gut-brain axis (95). Gut microbiota composition significantly influences intestinal serotonin production. Specific commensal bacteria, particularly those producing short-chain fatty acids (SCFAs) like butyrate, stimulate EC cells to synthesize and release serotonin (96, 97). Malnutrition-induced dysbiosis, characterized by a reduction in these beneficial SCFA-producing bacteria, can therefore lead to diminished serotonin synthesis in the gut (98).

Studies have found a strong link between abnormal gut microbiota composition and the onset of autism, depression, schizophrenia, and Alzheimer's disease (99, 100). An increasing body of evidence suggests that gut microbiota communicates with the central nervous system and can influence brain function and behavior through neural, endocrine, and immune pathways (101). For example, gut microbiota dysbiosis can lead to abnormal synthesis and release of neurotransmitters such as gamma-aminobutyric acid (GABA), and, as discussed, impair gut serotonin production. Dysbiosis can also induce neuroinflammation, disrupt the balance of brain neurotransmitters (including central serotonin levels, which may be influenced by peripheral precursor availability and signaling), and ultimately promote the occurrence of PND (102). The reduction in gut-derived serotonin and its downstream effects on central serotonergic signaling and neuroinflammation represent a significant pathway linking malnutrition, microbiota disruption, and impaired mental health/PND risk.

Animal studies suggest that surgical anesthesia can change the gut microbiome and impair cognitive function in elderly mice, and probiotic pretreatment can prevent the negative impact of surgical anesthesia on postoperative memory (103). Probiotics may exert part of their beneficial effect by restoring microbial communities that support healthy serotonin production and gut-brain signaling.

#### 4.2.4 Lymphatic system dysfunction

In the central nervous system, lymphatic drainage relies on the meningeal lymphatics in the dura mater and the glymphatic system, which interact to ensure the clearance of neurotoxic substances and the stability of the brain's microenvironment. Malnutrition impairs the lymphatic system's drainage function (104), leading to the accumulation of neuroinflammatory factors and metabolic products, thereby affecting neurocognitive function. Additionally, under conditions such as anesthetic drugs, surgical trauma, and changes in the patient's internal environment, immune imbalance in the brain can also lead to postoperative cognitive dysfunction. Lymphatic clearance is closely related to the sleep-wake cycle, with sleep promoting faster clearance of metabolites (105). Melatonin, a hormone that regulates the circadian rhythm and sleep-wake cycle and enhances sleep, is found in fruits and nuts, and its intake may be reduced in patients with malnutrition (106). Meanwhile, Mg^2+^, as one of the key elements in the human body, ensures sleep quality and regulates the body's circadian rhythm (107), and its deficiency may indirectly affect lymphatic clearance efficiency through sleep quality. Furthermore, sleep disruption increases neuronal activity and produces more waste, including lactate, which is then output through the lymphatic fluid (108).

#### 4.2.5 Endocrine disruption

**Figure 3:**
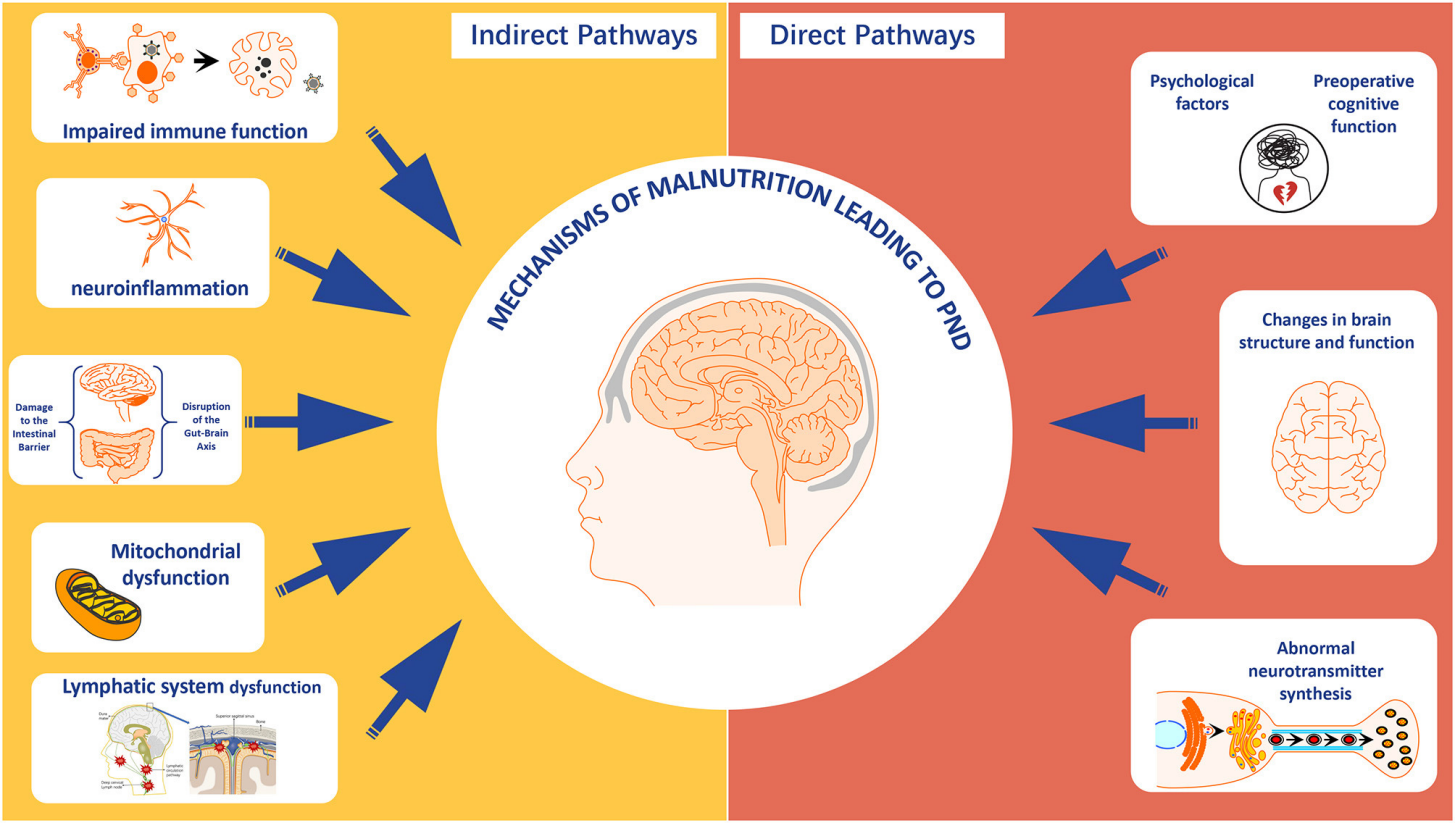
The direct and indirect mechanisms of malnutrition leading to perioperative neurocognitive disorder. Created with FigDraw.com.

Malnutrition can trigger functional disorders in the hypothalamic-pituitary-target gland axis, with the thyroid and adrenal axes being the most typical. Thyroid hormones play a crucial role in brain development, neuronal excitation, and metabolic rate. Malnutrition affects the synthesis and function of thyroid hormones due to insufficient dietary and trace element intake, thereby affecting cognitive function (109, 110). Additionally, patients with malnutrition have a disorder in the secretion of adrenocortical hormones under stress, leading to oxidative stress responses, neuronal damage, and promoting the occurrence of PND (Figure 3).

## 5 Nutritional intervention and PND

### 5.1 Immunonutrition

Current evidence supports the clinical benefits of immunonutrition formulas (containing arginine, nucleotides, and ω-3 fatty acids) in gastrointestinal and head/neck cancer surgeries. Two meta-analyse (111, 112) demonstrated that preoperative immunonutrition administration for 5–7 days significantly reduces the risk of postoperative infectious complications (RRs 0.71–0.74) and shortens hospital length of stay (MDs −1.22 to −2.12 days). These effects may be attributed to the mitigation of systemic inflammatory responses. Although direct evidence linking immunonutrition to delirium prevention remains sparse, the reduction in infectious complications could indirectly ameliorate neuroinflammation via decreased pro-inflammatory cytokine release (e.g., IL-6 and TNF-α). Emerging research highlights the gut microbiota-brain axis as a potential mechanistic pathway, where immunonutrition-derived short-chain fatty acids modulate blood-brain barrier permeability and neuroinflammatory microenvironments (113). Future studies should investigate longitudinal gut microbial dynamics in relation to neuroinflammatory biomarkers (e.g., CSF GFAP, neurofilament light chain) and optimize immunonutrition protocols within Enhanced Recovery After Surgery (ERAS) frameworks. Key considerations include dose-response relationships, timing of administration, and potential synergies/antagonisms with anti-inflammatory therapies.

### 5.2 Oral nutritional supplements (ONS)

For malnourished patients (NRS-2002 ≥3), preoperative ONS improves nutritional parameters (e.g., albumin and prealbumin levels) and reduces postoperative morbidity. High-biological-value protein formulations (e.g., whey protein) enhance nitrogen retention and hepatic protein synthesis (114, 115), with a meta-analysis revealing a 12.5% increase in prealbumin (95% CI: 5.3–19.7%; *P* < 0.01) following 7-day preoperative supplementation. Arginine/ω-3-enriched formulations further suppress NF-κB-mediated pro-inflammatory cascades (IL-6↓34%, TNF-α↓28%) while enhancing lymphocyte proliferation (113). A Cochrane review (116) confirmed ONS efficacy in reducing infectious complications in malnourished subgroups (RR = 0.58), though no mortality benefit was observed. Notably, evidence regarding cognitive outcomes remains inconclusive, warranting exploration of neuroprotective formulations incorporating choline, antioxidants (e.g., vitamin E, selenium), or omega-3 derivatives. Clinical implementation requires vigilant metabolic monitoring to avoid hyperglycemia and refeeding syndrome, ideally coordinated by multidisciplinary nutrition support teams (NSTs) for dynamic protocol adjustments (117).

### 5.3 Micronutrient optimization

Observational studies consistently associate vitamin D and B-complex deficiencies with POD risk. A retrospective cohort study in arthroplasty patients (118) demonstrated that post-operative vitamin D surveillance and supplementation improved bone turnover markers, though cognitive implications require validation. Iron homeostasis dysregulation (common post-gastrectomy) may exacerbate delirium via cerebral hypoxia and mitochondrial dysfunction (115), necessitating targeted repletion strategies. Prospective trials should evaluate multi-micronutrient cocktails modulating neuroinflammatory pathways (e.g., NLRP3 inflammasome inhibition) while addressing surgical subtype-specific deficiencies (e.g., vitamin B12 malabsorption after gastric resection).

### 5.4 Combined nutritional intervention and exercise training

Researchers have conducted several intervention studies to evaluate the effectiveness of combining nutritional improvement with exercise training in POD. A single-center study in the Netherlands investigated the use of multimodal prehabilitation to reduce the incidence of POD after elective abdominal surgery. The 5-week prehabilitation program, which included nutritional counseling and home-based exercise, reduced the incidence of POD by one-third (119). Another retrospective cohort study involving over 160,151 patients undergoing hip/femur surgery examined the use of early nutritional intervention (initiated on postoperative day 1) in malnourished patients. Although nutritional intervention was underutilized, its implementation was associated with reduced LOS without increasing hospital costs. However, the intervention did not improve secondary outcomes such as infection rates, in-hospital mortality, or ICU admission rates (120). While some randomized controlled trials (RCTs) suggest that appropriate nutrition can help reverse frailty and increase muscle mass index (121), a meta-analysis by Oktaviana et al. (122) of eight studies found that protein supplementation alone did not significantly improve muscle mass or frailty indices in frail populations. However, more promising results emerged when protein supplementation was combined with exercise intervention. A meta-analysis of 22 RCTs demonstrated significant improvements in frailty status, lean mass, muscle strength, and physical performance among frail older adults (123). Further research is needed to establish effective interventions for frail populations. Specifically, studies should investigate whether multimodal approaches combining exercise modalities—such as aerobic exercise, resistance training, balance training, and functional training—can enhance cardiopulmonary function, muscle strength, and muscle mass, thereby positively influencing perioperative cognitive function.

## 6 Research direction

In summary, as awareness of the impact of malnutrition continues to grow, interventions such as enteral nutrition (124), parenteral nutrition (125), and oral nutritional supplements (126) have been evaluated to explore the effect of nutritional improvement on preventing perioperative neurocognitive disorders. Moving forward, more targeted interventions tailored to different types of malnutrition are needed. For patients with metabolic obesity, the primary goal should be to alleviate chronic inflammation rather than achieving short-term weight loss (rapid weight loss before surgery may accelerate catabolism). Therefore, the future research direction should focus on how to improve the dietary structure and supplement antioxidant substances to reduce oxidative stress, regulate the activity of inflammasomes, inhibit the NF-κB pathway, and alleviate neuroinflammation. For patients with frail sarcopenia, the core goal is to reverse protein synthesis resistance and protect neuromuscular function. A single nutritional intervention strategy may be limited. Therefore, in the future, large-sample clinical trial evidence is needed to prove that a multi-dimensional collaborative approach integrating nutrition, exercise, metabolic regulation, and cognitive intervention may be beneficial for this population.

In addition, future research must prioritize elucidating how distinct malnutrition subtypes (undernutrition, specific micronutrient deficiencies and general malnutrition) contribute differentially to PND through discrete pathophysiological mechanisms. Current knowledge gaps center on two critical areas: (1) the cellular-level effects of nutrients on neuronal metabolism and neuroimmune crosstalk, and (2) subtype-specific pathways linking nutritional deficits to PND pathogenesis. To address these gaps, investigations should focus on subtype-targeted mechanisms:

For undernutrition, determine whether amino acid supplementation (e.g., tryptophan) restores serotonin synthesis in energy-deprived neurons to prevent delirium, and elucidate how protein-calorie deficits impair hippocampal neurogenesis.

For micronutrient deficiencies, test if vitamin B12 repletion mitigates neuroinflammation by preventing myelin degradation (contrasted with folate's role in dopamine methylation), and assess how zinc deficiency disrupts blood-brain barrier integrity via matrix metalloproteinase activation.

For general malnutrition, map synergistic effects of combined macro/micronutrient deficits on microglial activation, and characterize gut-brain axis dysregulation in patients with concurrent weight loss and vitamin depletion.

Collectively, these mechanistic insights will establish a foundation for precision nutritional interventions tailored to specific malnutrition phenotypes in clinical practice.

## 7 Conclusion

The review highlights a significant association between malnutrition and PND, with malnutrition increasing PND incidence and impacting postoperative recovery and quality of life. Mechanisms linking malnutrition to PND include psychological impacts, neurotransmitter synthesis abnormalities, brain structural and functional changes, immune dysfunction, neuroinflammation, mitochondrial issues, intestinal barrier damage, gut-brain axis disruption, lymphatic system dysfunction, and endocrine imbalances. Future research should delve into the efficacy of nutritional interventions in specific populations and the molecular links between nutrition and PND prevention, with studies on gene expression, neurotransmitter metabolism, and inflammatory modulation potentially informing more targeted and potent clinical nutrition strategies.
